# Care Dependency in Non-Hospitalized Patients with COVID-19

**DOI:** 10.3390/jcm9092946

**Published:** 2020-09-12

**Authors:** Anouk W. Vaes, Felipe V.C. Machado, Roy Meys, Jeannet M. Delbressine, Yvonne M.J. Goertz, Maarten Van Herck, Sarah Houben-Wilke, Frits M.E. Franssen, Herman Vijlbrief, Yvonne Spies, Alex J. Van ’t Hul, Chris Burtin, Daisy J.A. Janssen, Martijn A. Spruit

**Affiliations:** 1 Department of Research and Development, Ciro, 6085 NM Horn, The Netherlands; felipemachado@ciro-horn.nl (F.V.C.M.); roymeys@ciro-horn.nl (R.M.); jeannetdelbressine@ciro-horn.nl (J.M.D.); yvonnegoertz@ciro-horn.nl (Y.M.J.G.); maartenvanherck@ciro-horn.nl (M.V.H.); sarahwilke@ciro-horn.nl (S.H.-W.); fritsfranssen@ciro-horn.nl (F.M.E.F.); daisyjanssen@ciro-horn.nl (D.J.A.J.); martijnspruit@ciro-horn.nl (M.A.S.); 2 Nutrim School of Nutrition and Translational Research in Metabolism, 6229 HX Maastricht, The Netherlands; 3 Department of Respiratory Medicine, Maastricht University Medical Centre (MUMC+), 6229 HX Maastricht, The Netherlands; 4 REVAL—Rehabilitation Research Center, BIOMED—Biomedical Research Institute, Faculty of Rehabilitation Sciences, Hasselt University, 3500 Diepenbeek, Belgium; chris.burtin@uhasselt.be; 5 Lung Foundation Netherlands, 3818 LE Amersfoort, The Netherlands; hermanvijlbrief@longfonds.nl (H.V.); yvonnespies@longfonds.nl (Y.S.); 6 Department of Pulmonary Disease, Radboud University Medical Center, 6525 GA Nijmegen, The Netherlands; Alex.vantHul@radboudumc.nl; 7 Department of Health Services Research, Care and Public Health Research Institute, Faculty of Health, Medicine and Life Sciences, Maastricht University, 6229 ER Maastricht, The Netherlands

**Keywords:** COVID-19, care dependency, activities of daily living

## Abstract

Background: A large sample of “mild” COVID-19 patients still experience multiple symptoms months after being infected. These persistent symptoms are associated with many clinically relevant outcomes, including poor health status and impaired functional status. To date, no information is available about care dependency. Therefore, we aimed to explore the level of care dependency and the need for assistance with personal care in non-hospitalized COVID-19 patients. Methods: Members of two Facebook groups for COVID-19 patients with persistent complaints in The Netherlands and Belgium, and from a panel of people who registered at a website of the Lung Foundation Netherlands, were assessed for demographics, pre-existing comorbidities, health status, and symptoms. In addition, patients were asked about their dependence on others for personal care before and after the infection. The level of care dependency was assessed with the Care Dependency Scale (CDS) in members of the Belgian Facebook group (*n* = 210). Results: The data of 1837 non-hospitalized patients (86% women; median (IQR) age: 47 (38–54)) were analyzed. Only a small proportion of patients needed help with personal care before COVID-19, but the care need increased significantly after the infection (on average 79 ± 17 days after the onset of symptoms; 7.7% versus 52.4%, respectively; *p* < 0.05). The patients had a median (IQR) CDS score of 72 (67–75) points, and 31% of the patients were considered as care-dependent (CDS score ≤ 68 points). Conclusions: COVID-19 has an important impact on care dependency in non-hospitalized patients. About three months after the onset of symptoms, a considerable proportion of non-hospitalized patients were to some degree dependent on others for personal care. This indicates that the impact of COVID-19 on patients’ daily lives is tremendous, and more attention is needed to identify optimal treatment strategies to restore patients’ independency.

## 1. Introduction

Since the beginning of the worldwide outbreak of severe acute respiratory syndrome coronavirus-2 (SARS-CoV-2), most studies have focused on hospitalized patients with severe coronavirus disease 2019 (COVID-19). Recently, there is an increasing awareness of the majority of patients (>80%), in which the illness is relatively mild and can be self-managed without hospital admission [1,2]. Goërtz and colleagues were the first to highlight the unmet healthcare needs of a large group of non-hospitalized COVID-19 patients [3]. They demonstrated that about three months after the infection, a large sample of COVID-19 patients still experienced multiple symptoms, providing evidence for a post-COVID-19 syndrome [3]. These persistent symptoms should be taken seriously, as these showed a clear association with many clinically relevant outcomes, including a low quality of life [4]. It is reasonable to assume that the reported symptoms may also limit patients’ ability to perform activities of daily living (ADLs), including personal care (e.g., washing, dressing, toileting, eating). Recently, Belli and colleagues showed that about half of the elderly, multimorbid, and hospitalized COVID-19 patients reported severe problems with the performance of ADLs at discharge to home [5]. To date, no information is available about care dependency in middle-aged, non-hospitalized COVID-19 patients who reported good health before the infection [3]. Therefore, the aim of this study was to explore the level of care dependency in ADLs and the need for assistance with personal care in non-hospitalized COVID-19 patients. 

## 2. Materials and Methods

### 2.1. Study Design and Participants

As part of a large cross-sectional study conducted in The Netherlands and Flanders (Belgium), an online questionnaire was made available between 4 June and 11 June 2020 to all members of two Facebook groups for coronavirus patients with persistent complaints in The Netherlands (~11,000 members: “Corona ervaringen en langdurige klachten!”) [6] and Flanders (~1200 members: “Corona patiënten met langdurige klachten (Vlaanderen)”) [7], and to a panel of ~1200 people who registered at a website of the Lung Foundation Netherlands (www.coronalongplein.nl) for additional information regarding COVID-19. The medical ethics committee of Maastricht University stated that the Medical Research Involving Human Subjects Act (WMO) did not apply for this study, and that an official approval of this study by the committee was not required (METC2020-1978). The medical ethics committee of Hasselt University formally judged and also approved the study (MEC2020/041). All the respondents gave digital informed consent at the start of the questionnaire. Without the informed consent, the remaining questionnaire could not be completed. The study was registered before its start (trialregister.nl: NL8705). Data on symptom burden and its association with clinically relevant outcomes have been published before [3].

### 2.2. Measures

The survey contained questions regarding demographics, pre-existing comorbidities, COVID-19 diagnosis (based on reverse transcription polymerase chain reaction (RT-PCR) test and/or a computed tomography (CT) scan of the thorax; symptom-based medical diagnosis; no test/medical diagnosis), intensive care unit (ICU) or hospital admission, and self-reported health status (good/moderate/poor). The symptom prevalence and intensity were measured using the Utrecht Symptom Diary (USD), which is an adapted Dutch version of the Edmonton Symptom Assessment System (ESAS) [8]. The USD assesses 15 frequently occurring symptoms using an 11-point numerical rating scale ranging from 0 (no symptoms) to 10 points (worst intensity). Cut-off scores were used to stratify the symptoms into five categories: none (0 points), mild (1–3 points), moderate (4–6 points), severe (7–9 points), and very severe (10 points). In patients from the Belgian Facebook group, the level of care dependency after the infection was assessed using the Care Dependency Scale (CDS). The CDS consists of 15 items regarding basic and instrumental ADLs, such as personal care, household activities, and social and recreational activities [9]. A 5-point Likert scale assesses the patients’ reliance on each item, from completely dependent to completely independent. The total score ranges from 15 (worst) to 75 points (best). Patients with a CDS score of ≤68 points are considered as care-dependent [10]. Using this cut-off results in a sensitivity of 0.85 and a positive predictive value of 0.90 for detecting care dependency [10]. In addition, all the patients were asked about their dependence on others (partner/family/other) for personal care before and after the infection.

### 2.3. Statistics

Data were presented as mean and standard deviation (SD), median and interquartile range (IQR), or frequencies and proportions, as appropriate. Between-group analyses were performed with a Chi square test or Mann–Whitney U Test. The differences within-groups were evaluated with the McNemar Test or Wilcoxon rank sum test. Statistical analyses were performed using SPSS version 25.0. A priori, the level of significance was set at *p* < 0.05; analyses were corrected for multiple comparisons using Bonferroni correction.

## 3. Results

A total of 2159 patients responded to the online questionnaire (estimated response rate: 16%). Data from 220 respondents were excluded, as they were admitted to an ICU (*n* = 15), gender was not reported (*n* = 9), the onset of symptoms was before 1 January 2020 (the first official confirmed case of COVID-19 in The Netherlands was on 28 February 2020, and in Belgium on 4 February 2020; *n* = 8), the onset of symptoms was less than three weeks ago (*n* = 14), or the survey was incomplete (*n* = 174). In addition, data from 102 hospitalized patients were excluded. 

Finally, the data of 1837 non-hospitalized patients (86% women; median age (IQR): 47 (38–54) were used for analyses (Table 1).

Seventy-one percent of the patients were married or living with a partner, and slightly more than half of the patients had children living at home. Three hundred and nineteen patients (17%) had a confirmed diagnosis of COVID-19 based on RT-PCR/CT testing, 820 patients (45%) were medically diagnosed with a high suspicion of COVID-19 based on their symptoms, and 698 patients (38%) were undiagnosed at the start of the presumed infection.

Eighty-six percent of the patients reported a good health status before the infection, and only 12 patients reported a poor health status. After the infection (on average 79 ± 17 days after onset of symptoms), there was a significant decrease in self-reported health status; only 6% of the patients had a good health status, while the majority of the patients reported a moderate (64%) or poor (30%) health status. Moreover, a high proportion of patients experienced long-term symptoms, including fatigue (98%), muscle weakness (90%), sleeping problems (88%), and pain (87%).

**Figure 1:**
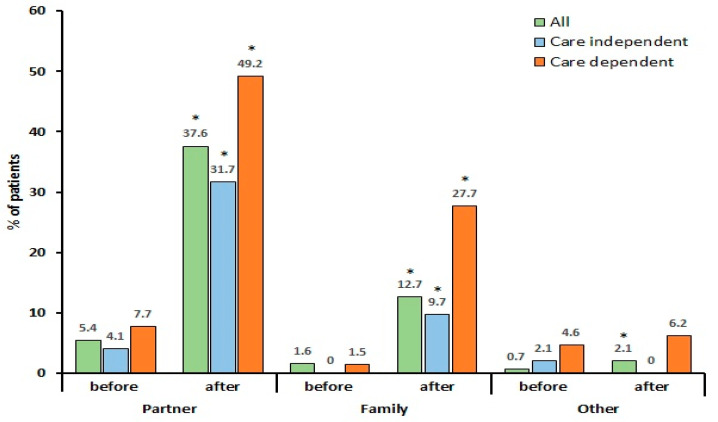
Need for help with personal care before and after coronavirus disease 2019. * *p* < 0.05 versus before infection based on the McNemar test. All (*n* = 1837); care-dependent: Care Dependency Scale (CDS) score ≤ 68 points (*n* = 65); care independent: CDS score > 69 points (*n* = 145).

Only a small proportion of patients needed help with personal care before the COVID-19 infection, but care needs increased significantly after the infection. Indeed, at the time of completing the questionnaire, more than half of the patients were care-dependent on their partner, family, or others (Figure 1).

### Level of Care Dependency

**Figure 2:**
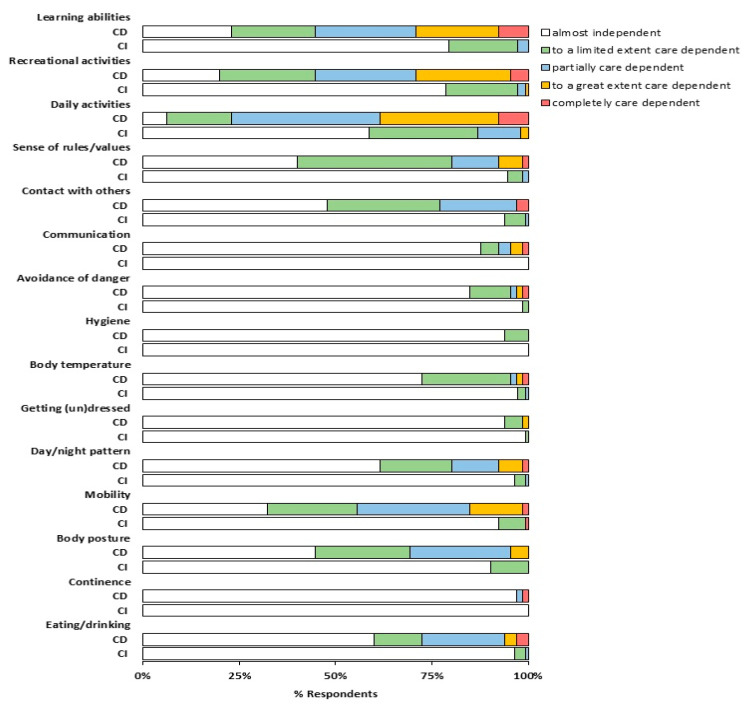
Care Dependency Scale (CDS) item scores for care-dependent (CD) and care-independent (CI) patients; differences in the proportion of patients across different item scores were compared using the McNemar test; care-dependent: CDS score ≤ 68 points; *n* = 65; care-independent: CDS score > 69 points *n* = 145.

The level of care dependency was determined using the CDS in a subgroup of 210 Belgian patients (88% women; median age (IQR): 44 (37–52) years) (Table 1). After the infection, the patients had a median (IQR) CDS score of 72 (67–75) points. Sixty-five patients (31%) had a CDS score of ≤68 points and were considered as care-dependent. The care-dependent patients scored significantly lower on all the CDS items, except continence, compared to the care-independent patients (Figure 2). The highest differences were found for mobility, recreational activities, and learning abilities, in which 67.7%, 80%, and 76.9% of the care-dependent reported to be at least to some extent care-dependent versus 7.6%, 21.4%, and 20.7% of the care-independent patients, respectively (all *p* < 0.001). Interestingly, 41.1% of the care-independent patients reported to be at least to a limited extent dependent in the performance of daily activities (Figure 2).

**Figure 3:**
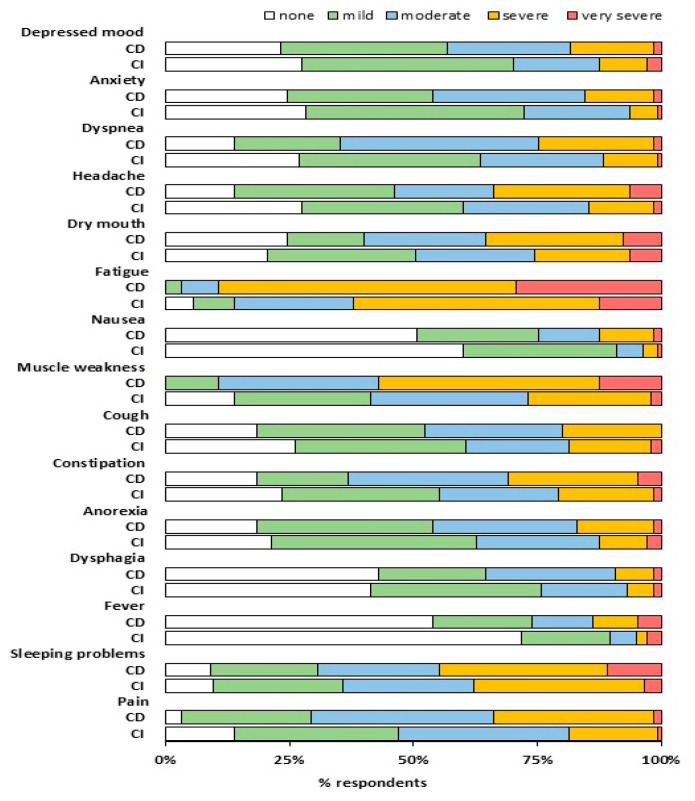
Symptom intensity for care-dependent (CD) and care-independent (CI) patients; care-dependent: Care Dependency Scale (CDS) score ≤ 68 points (*n* = 65); care-independent: CDS score > 69 points (*n* = 145). Symptom intensity based on Utrecht Symptom Diary score: none: 0 point; mild: 1–3 points; moderate: 4–6 points; severe: 7–9 points; and very severe: 10 points. Differences in the proportion of patients across different symptom intensity categories between the CD patients and CI patients were tested with the McNemar test.

The care-dependent patients were significantly younger and had a worse health status before the infection compared to the care-independent patients, while the proportion of patients reporting to need help with personal care before the infection was comparable (Table 2). The majority of the patients perceived support from family regularly to very often, which was not different between the care-dependent patients and the care-independent patients (72.3% versus 64.1%, respectively). Both groups reported a significant decrease in health status and an increased need for help with personal care after the infection (Table 2, Figure 1), however the health status was worse in the care-dependent patients, and they also needed help more often compared to the care-independent patients. Furthermore, the care-dependent patients had a higher prevalence of mild to very severe fatigue (100.0% vs. 94.5%), muscle weakness (100.0% vs. 86.2%), pain (96.9% vs. 86.2%), dyspnea (86.2% vs. 73.1%), headache (86.2% vs. 72.4%), and fever (46.2% vs. 28.3%) (all *p* < 0.05) (Figure 3).

## 4. Discussion

This is the first study exploring the level of care dependency in non-hospitalized COVID-19 patients with persistent complaints about three months after the onset of COVID-related symptoms. About half of these patients have increased care needs. Moreover, based on the CDS score, one third of the patients were considered as care-dependent in ADLs, including personal care and household activities. Again, these data demonstrate the major impact of COVID-19 on daily functioning, even months after the infection in a relatively young and healthy sample of patients.

Rehabilitation guidelines for COVID-19 patients after discharge from the hospital already recognize the increased care dependency of patients and advise to include functional therapy (e.g., physiotherapy and occupational therapy) for improving ADLs and to facilitate functional independence [11]. However, no attention has been given to the group of non-hospitalized patients, while our results clearly demonstrated that these patients experience serious problems in the performance of ADLs and do require assistance from their partner and family with personal care. Interestingly, the increased need for help with personal care after the infection was not only seen in the care-dependent patients, but also in the patients that were considered as care-independent (from 6% of the patients before to 41% of the patients after the infection; *p* < 0.05). As the ADLs of these non-hospitalized patients are often not assessed, they might not receive the most optimal treatment.

Earlier studies in community-dwelling elderly, nursing home patients, and patients with chronic diseases (i.e., advanced heart failure, renal failure, or respiratory disease) already demonstrated that increased care dependency may lead to frustration, social isolation, a poorer quality of life, and an increased risk of mortality [12,13,14,15,16], which underlines the importance of assessing the care needs in COVID-19 patients. In this, the perceived family support is valuable, as this is positively associated with an improved health-related quality of life, ADL performance, and self-care behavior [17,18]. However, the increased care needs of the patients may impose a high burden on the informal caregivers. Findings in patients with chronic obstructive pulmonary disease (COPD) already demonstrated that informal caregivers may experience anxiety and depression, worries about the patient, uncertainty about the future, social isolation, and occupation-related problems [19,20]. As informal care givers of COVID-19 patients are often still employed and may have insufficient knowledge about the required health-care duties, they may be expected to experience increased psychological stress [21].

Thirty-one percent of the non-hospitalized patients were considered as care dependent about three months after the infection (CDS score ≤ 68 points). Strikingly, the CDS scores in our study were comparable to earlier findings in patients with advanced chronic renal failure [22], while the scores of care-dependent patients were even worse than reported in patients with end-stage COPD or chronic heart failure, and elderly living in residential care homes in The Netherlands [15,23,24]. This indicates that the impact of COVID-19 on patients’ daily life is tremendous in a subset of non-hospitalized patients, and more attention should be given to identify optimal treatment options for these patients, including occupational therapy.

While increasing age is known to be a key determinant of care dependency [25], it is notable that most of the patients included in this study were only middle-aged, but still reported high levels of care dependency. The care dependent patients were even younger compared to the care-independent patients. The increased need for assistance with ADLs might at least in part be explained by the higher symptom burden in care-dependent patients. On average, care-dependent patients experienced symptoms more frequently and the symptom intensity was higher compared to care-independent patients. It is reasonable to assume that these symptoms, including fatigue, muscle weakness, dyspnea, and pain, may negatively affect the self-reliance and ability to perform ADLs. Then again, the majority of patients are highly symptomatic, but not care-dependent. So, besides the high symptom burden, other factors may play a role in becoming care-dependent. For example, care dependent patients had a worse self-reported health status before the infection, which is known to be a powerful indicator for care dependency [26]. Furthermore, the tendency towards a higher prevalence of multimorbidity in care-dependent patients may contribute to an increased risk of becoming care-dependent [27].

The following methodological considerations need to be considered. First, this study also included patients with suspected COVID-19, and 116 patients reported symptoms in the period between 1 January 2020 and the first confirmed infection in either The Netherlands or Belgium, of which 58.6% were undiagnosed. As the COVID-19 testing capacity was too limited, not all patients were tested in the beginning of the pandemic. Secondary analyses including only patients with a confirmed diagnoses of COVID-19 yielded similar results. Then again, Greenhalgh and colleagues already defined post-acute COVID-19 as extending beyond three weeks from the onset of first symptoms and chronic COVID-19 as extending beyond 12 weeks [28]. Since many people were not tested, and false negative tests are common, it was suggested that a positive test for COVID-19 is not a prerequisite for diagnosis [28]. We demonstrated that a subgroup of non-hospitalized and non-tested patients with suspected COVID-19 may still experience serious symptoms and limitation in ADLs months after the infection and are below the radar of healthcare professionals. This group, however, requires further attention. Indeed, the proportion of patients with confirmed COVID-19 was similar between the care-dependent and independent groups. Second, our study sample is not representative for all COVID-19 patients. Sampling bias cannot be ruled out, as patients exposed to the survey and willing to participate in this study are probably the ones experiencing long-term symptoms and limitations in ADL performance. Moreover, as there is a disproportionate distribution regarding gender, caution should be used in generalizing the findings. Third, in this study we used a self-reported tool to assess care dependency instead of objective measures of functional status [29]. Then again, the CDS is easy to use, takes less than 5 min to complete, and has shown to be a reliable and valid tool for assessing care dependency in several patient categories in different settings [9,30]. Moreover, the CDS is not limited to the basic activities of daily living, but provides information on a broad range of patient care needs, including learning abilities and the ability for social and recreational activities [9].

To date, long-term data from COVID-19 survivors are lacking. However, our findings and data on symptom burden and its association with clinically relevant outcomes suggest that post-COVID syndrome will have a serious impact on healthcare systems and global society. Indeed, many patients with persistent symptoms reported a loss of work productivity and will need additional healthcare. Therefore, more attention should be given to better understand the care needs and persistent symptoms in this highly heterogeneous group of post-COVID-19 patients, and to identify possible predictive markers for the post-COVID-19 syndrome.

To conclude, our findings showed that COVID-19 has an important impact on care dependency in non-hospitalized patients. About three months after the onset of symptoms, a considerable proportion of patients with persistent complaints was to some degree dependent on others for personal care and the performance of ADLs. Further studies should focus on better profiling individual care needs and optimizing treatment strategies for this highly underexposed group of COVID-19 patients.

## Figures and Tables

**Table 1:** Patient characteristics.

Variables		All Patients (*n* = 1837)		Belgium Facebook Group (*n* = 210)	
Gender—female, *n* (%)		1581 (86.1)		184 (87.6)	
Age, years ^a^		47.0 (38.0–54.0)		44.0 (37.0–52.3)	
BMI, kg/m^2 a^		25.1 (22.5–28.7)		25.1 (22.2–28.6)	
Marital status—married/living with partner, *n* (%)		1308 (71.2)		147 (70.0)	
Children living at home, yes, *n* (%)		949 (51.7)		118 (56.2)	
1		310 (32.7)		30 (25.4)	
2		480 (50.6)		69 (58.5)	
3		131 (13.8)		14 (11.9)	
≥4		28 (2.9)		5 (4.2)	
Comorbidities before COVID-19, *n* (%)	None	1132 (61.6)		129 (61.4)	
	1	465 (25.3)		49 (23.3)	
	≥2	240 (13.1)		32 (15.2)	
Health status, *n* (%)		Before infection	After infection	Before infection	After infection
Good		1576 (85.8)	117 (6.4) *	175 (83.3)	15 (7.1) *
Moderate		249 (13.6)	1170 (63.7) *	35 (16.7)	137 (65.2) *
Poor		12 (0.7)	550 (29.9) *	0.0	58 (27.6) *

BMI = body mass index; kg = kilogram; m = meter; ^a^ median (IQR); * *p* < 0.05 vs. before infection.

**Table 2:** Patient characteristics stratified for level of care dependency.

		CDS Score ≤ 68 Points (*n* = 65)		CDS Score > 69 Points (*n* = 145)	
Gender—female, *n* (%)		56 (86.2)		128 (88.3)	
Age, years ^a^		41.0 (33.5–51.0)		45.0 (38.5–53.0) *	
BMI, kg/m^2 a^		25.1 (21.9–29.0)		25.1 (22.5–28.5)	
Marital status—married/living with partner, *n* (%)		45 (69.2)		102 (70.3)	
Children living at home, yes, *n* (%)		29 (44.6)		89 (61.4) *	
1		9 (31.0)		21 (23.6)	
2		14 (48.3)		55 (61.8)	
3		3 (10.3)		11 (12.4)	
≥4		3 (10.3)		2 (2.2)	
Comorbidities before COVID-19, *n* (%)	None	39 (60.0)		90 (62.1)	
	1	13 (20.0)		36 (24.8)	
	≥2	13 (20.0)		19 (13.1)	
COVID-19 Diagnosis					
Based on CT/RT-PCR testing, *n* (%)		15 (23.1)		34 (23.4)	
Based on symptoms, *n* (%)		32 (49.2)		73 (50.3)	
Undiagnosed, *n* (%)		18 (27.7)		38 (26.2)	
Health status before, *n* (%)		Before infection	After infection	Before infection	After infection
Good		48 (73.8)	1 (1.5) ^#^	127 (87.6) *	14 (9.7) *^,#^
Moderate		17 (26.2)	29 (44.6) ^#^	18 (12.4) *	108 (74.5) *^,#^
Poor		-	35 (53.8) ^#^	-	23 (15.9) *^,#^
Need for help with personal care, *n* (%)		Before infection	After infection	Before infection	After infection
Partner		5 (7.7)	32 (49.2) ^#^	6 (4.1)	46 (31.7) *^,#^
Family		1 (1.5)	18 (27.7) ^#^	0.0	14 (9.7) *^,#^
Nurse		1 (1.5)	2 (3.1)	0.0	0.0 *
Other		2 (3.1)	2 (3.1)	3 (2.1)	0.0 *
Perceived support from family, *n* (%)					
Very often		26 (40.0)		45 (31.0)	
Regularly		21 (32.3)		48 (33.1)	
Occasionally		13 (20.0)		44 (30.3)	
Rarely or never		4 (6.2)		6 (4.1)	
Not applicable		1 (1.5)		2 (1.4)	
CDS, sum score ^a^		64.0 (61.5–67.0)		74.0 (72.0–75.0) *	

CDS = Care Dependency Scale; BMI = body mass index; kg = kilogram; m = meter; CT/RT-PCR = reverse transcription polymerase chain reaction/computed tomography. ^a^ median (IQR); * *p* < 0.05 vs. CDS score ≤ 68 points; ^#^ *p* < 0.05 vs. before infection.
